# Thermogravimetric Analysis Properties of Cellulosic Natural Fiber Polymer Composites: A Review on Influence of Chemical Treatments

**DOI:** 10.3390/polym13162710

**Published:** 2021-08-13

**Authors:** N. M. Nurazzi, M. R. M. Asyraf, M. Rayung, M. N. F. Norrrahim, S. S. Shazleen, M. S. A. Rani, A. R. Shafi, H. A. Aisyah, M. H. M. Radzi, F. A. Sabaruddin, R. A. Ilyas, E. S. Zainudin, K. Abdan

**Affiliations:** 1 Institute of Tropical Forestry and Forest Products (INTROP), Universiti Putra Malaysia (UPM), Seri Kembangan 43400, Selangor, Malaysia; mohd.nurazzi@gmail.com (N.M.N.); shazra.shazleen@yahoo.com (S.S.S.); ayu.rafiqah@yahoo.com (A.R.S.); hafiezalradzi@unimap.edu.my (M.H.M.R.); 2 Centre for Defence Foundation Studies, Universiti Pertahanan Nasional Malaysia (UPNM), Kem Perdana Sungai Besi, Kuala Lumpur 57000, Malaysia; 3 Department of Aerospace Engineering, Universiti Putra Malaysia (UPM), Seri Kembangan 43400, Selangor, Malaysia; asyrafriz96@gmail.com; 4 Faculty of Science, Universiti Putra Malaysia (UPM), Seri Kembangan 43400, Selangor, Malaysia; marwahrayung@yahoo.com; 5 Research Centre for Chemical Defence, Universiti Pertahanan Nasional Malaysia (UPNM), Kuala Lumpur 57000, Malaysia; faiznorrrahim@gmail.com; 6 School of Materials and Minerals Resources Engineering, Engineering Campus, Universiti Sains Malaysia, Nibong Tebal 14300, Penang, Malaysia; saifulasmal@gmail.com; 7 Department of Mechanical and Manufacturing Engineering, Faculty of Engineering, Universiti Putra Malaysia (UPM), Seri Kembangan 43400, Selangor, Malaysia; 8 Faculty of Engineering Technology, Universiti Malaysia Perlis, Padang Besar 02100, Perlis, Malaysia; 9 Faculty of Biotechnology and Biomolecular Sciences, Universiti Putra Malaysia (UPM), Seri Kembangan 43400, Selangor, Malaysia; 10 School of Chemical and Energy Engineering, Faculty of Engineering, Universiti Teknologi Malaysia (UTM), Skudai 81310, Johor, Malaysia; ahmadilyas@utm.my

**Keywords:** chemical treatment, natural fiber, polymer composites, TGA, thermal stability

## Abstract

Natural fiber such as bamboo fiber, oil palm empty fruit bunch (OPEFB) fiber, kenaf fiber, and sugar palm fiber-reinforced polymer composites are being increasingly developed for lightweight structures with high specific strength in the automotive, marine, aerospace, and construction industries with significant economic benefits, sustainability, and environmental benefits. The plant-based natural fibers are hydrophilic, which is incompatible with hydrophobic polymer matrices. This leads to a reduction of their interfacial bonding and to the poor thermal stability performance of the resulting fiber-reinforced polymer composite. Based on the literature, the effect of chemical treatment of natural fiber-reinforced polymer composites had significantly influenced the thermogravimetric analysis (TGA) together with the thermal stability performance of the composite structure. In this review, the effect of chemical treatments used on cellulose natural fiber-reinforced thermoplastic and thermosetting polymer composites has been reviewed. From the present review, the TGA data are useful as guidance in determining the purity and composition of the composites’ structures, drying, and the ignition temperatures of materials. Knowing the stability temperatures of compounds based on their weight, changes in the temperature dependence is another factor to consider regarding the effectiveness of chemical treatments for the purpose of synergizing the chemical bonding between the natural fiber with polymer matrix or with the synthetic fibers.

## 1. Introduction

The interest in producing natural fiber-reinforced polymer composites has gained considerable attention in the last few years due to sustainability and degradability issues [1]. Natural fibers have been introduced with a view to producing lightweight composites, along with reduced prices relative to conventional synthetic fibers such as fiber glass, carbon fiber, and Kevlar, as well as to produced green-reinforced polymer composites. Natural cellulose fibers have a wide range of physical and mechanical properties, which depend on the source of the fibers and their characteristics, such as diameter, length, specific gravity, etc., determining their application. The use of natural fibers such as kenaf fiber [2], sugar palm fiber [3], flax fiber [4,5], jute [6], OPEFB fiber [7], pineapple leaf fiber (PALF) [8], and banana fiber [9] in polymer composites have several advantages, including low density, availability, biodegradability, recyclability, increased physical and mechanical properties, and good thermal stability of the resulting composites [3]. Natural fiber (such as kenaf, jute, hemp)-reinforced polymer composites have outstanding and comparable thermal properties to synthetic fibers, leading to extend their applications for engineering materials such as automotive, aerospace industry, and construction structures [10,11,12,13,14]. However, before their applications in structural fields, some testing techniques are required to investigate the composite structure and performance under periodic stress and a certain level of the temperature effect.

**Figure 1:**
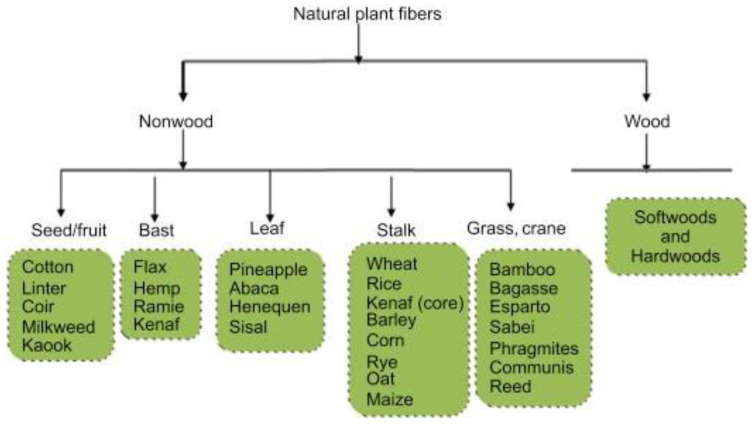
Classification of plant fibers based on their sources. Reproduced from ref. [15].

Natural fiber can be classified into two main groups based on its origins, namely inorganic and organic. Inorganic natural fibers are usually composed of mineral fibers such as asbestos, wollastonite, and fibrous brucite. Natural fibers can be categorized into two groups, which are animal fibers and plant fibers. Animal fibers are natural fibers made up mostly of proteins; thus they are commonly known as protein-based fibers, including wool, silk, hair, fur, and feathers. In comparison, plant fibers are those derived from plants and are generally referred to as cellulose-based fiber. The fibers are graded according to their location in the plant, such as stalk, stem, bast, leaf, or seed of the plant, as seen in Figure 1. Bast fibers include kenaf fiber, ramie fiber, flax fiber, and jute fiber, which is obtained by the retting process, where the stems are soaking in the water with or without chemicals or any substances. Fiber from leaves such as banana, pineapple, and abaca are separated through crushing and grinding. Other plants’ stalks, such as grass, bamboo, wheat, and bagasse, may even make fibers.

**Figure 2:**
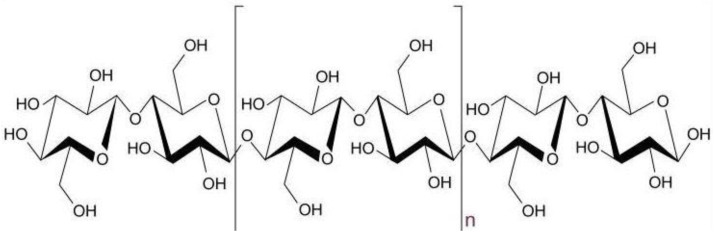
Cellulose structure which is a linear polymer made up of β-D-glucopyranose units covalently linked with (1–4) glycosidic bonds. Reproduced from ref. [20].

**Figure 3:**
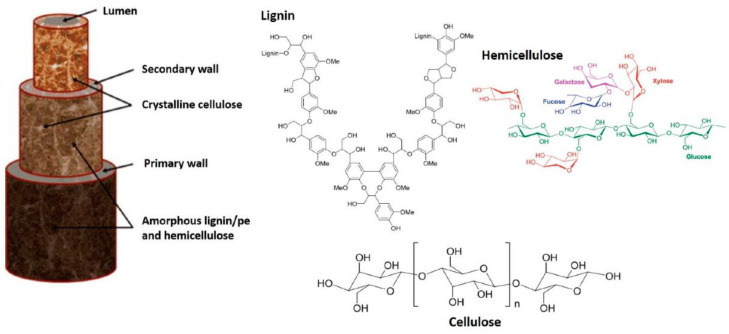
Schematic representation of the fibrillar structure of plant fibers. Reproduced from ref. [21].

Plant fibers are composed of three main components: namely, cellulose, hemicelluloses, and lignin. These fibers are also referred to as lignocellulosic materials, since cellulose is the main chemical component, with differing amounts of hemicellulose and lignin [16]. Cellulose is a linear semi-crystalline polysaccharide consisting of anhydroglucose units (AHU) linked by 1,4-β-glycoside bonds, which contain hydroxyl groups, as shown in Figure 2. These hydroxyl groups form intramolecular hydrogen bonds inside the macromolecule and intermolecular hydrogen bonds among other cellulose macromolecules. The cellulose structure is made up of crystalline and amorphous regions. Strong inter-molecular hydrogen bonds with large molecules are established by crystalline cellulose [17]. The hydrogen bonds between macromolecules, crystalline cellulose fibrils, and their orientation in the cell wall give the fiber its mechanical strength, as illustrated in Figure 3. Lignin is firmly attached to hemicellulose; it cross-links to polysaccharides and fills the gaps in the cell wall between the cellulose, hemicellulose, and pectin components, giving the cell wall strength. The functional groups for all three structures are susceptible to chemical alteration when utilizing treatments in the production process to make the fibers more compatible with the polymer matrix [18,19].

The main limitations of natural fibers are their hydrophilic properties, which may increase the absorption of moisture and deteriorate the bonding interaction between the fibers and the polymer matrices. The other limitations of natural fibers, such as their non-uniformity, poor thermal stability, and limited mechanical strength, also contributed to unfavorable properties relative to synthetic or manufactured reinforced composite [22,23,24,25]. These demerits, including the poor interfacial bonding and exhibition of the hydrophilic property of natural fiber, can be avoided through various chemical treatments that alter the compatibility between the natural fiber with the polymer matrix. These chemical treatments form fully cured and cross-linked natural fibers that reinforced the polymeric matrix to attain improved mechanical strength and thermal stability [26,27]. A better compatibility between natural fiber and its polymer matrix may occur when the surface impurities and hydroxyl group components, such as pectin, lignin, and hemicellulose, are effectively washed from the fiber [28]. This phenomenon forms a strong chemical bonding between two phases’ interfaces and a good network, and it can increase the applications of natural fiber, such as marine and automobile sectors, because the process increases the resistance to creep, wear, and tear [29,30,31,32]. The enhancement in these properties can improve the material’s service life, especially when exposed to extreme conditions, such as elevated temperature, high humidity, and acidic environment [33,34].

The properties of natural fibers affect the structural strength of the natural fiber-reinforced composites system. Thus, the efficiencies of the stress transfer mechanism from the matrix to the fibers were significant. Globally, natural fibers applications in composites are very limited to the interior parts in automotive industries due to their relatively lower mechanical properties and poor surface adhesion between the fibers and matrix [35]. The poor interfacial bonding between natural fibers and the matrix leads to the deterioration of the mechanical composites [36]. This is because natural fibers are hydrophilic and physicochemically heterogeneous, affecting their continuity and uniformity. The incompatibility of the hydrophilic nature of natural fibers with a hydrophobic matrix polymer leads to poor adhesion between those components, resulting in non-uniform spreading of the fibers inside the matrix. However, these drawbacks can be overcome through the applications of various types of surface treatment, chemicals, and coatings. Through these treatments, natural fibers enable overcoming the challenges in terms of moisture absorption, adhesion, fire resistance, strength, and weather-dependence, which varied their consistency. Natural fibers also have a complicated process of preparation. For example, sugar palm fiber is arbitrarily wrapped along the rib of palm leaves [37]. These properties affected its interaction with the polymer matrix when it is used directly. Sugar palm fiber also showed a good example of a fiber-oriented structure in the composites system. The long fiber of sugar palm fibers usually is tangled and random in orientation mixed and infused into the mold via the lay-up method. Jariwala and Jain [38] found short and arbitrarily organized sugar palm fibers to have low mechanical properties; thus, they are not suitable for structural application where mechanical efficiency is needed.

This article provides a state-of-the-art review on the influence of chemical treatments on the thermal performance of natural fiber polymer composites in various applications. Additionally, this review can be used as a guide to gathering information on fiber treatments (e.g., alkali, silane, and acetylation treatments) to extend the quality of the thermal stability of natural fiber-reinforced polymer composites for future outlooks.

## 2. Chemical Treatments of Natural Fiber

To meet the demand for environmentally friendly composite materials, researchers have focused on the use of natural fibers. Natural fibers have advantages over synthetic fibers due to their low cost, availability, biodegradability, and recyclability [39]. Thus, natural fibers are a green alternative to synthetic fibers as reinforcement for polymer composites. Apart from these benefits, natural fibers impart some drawbacks, including the incompatibilities with the matrix and high absorption of moisture. The presence of waxes and pectin in the cell wall of natural fibers covered the fibers’ reactive functional groups, thus preventing the interlocking with the matrix. The inaccessibility of fibers with matrix leads to poor adhesion across the phase boundary and weak dispersion of force and poor strength properties. The structural composition of natural fiber-like cellulose, hemicellulose, lignin, pectin, and wax substances allows the unstable effect caused by the moisture and weak adhesion with the polymer matrix [40,41]. Therefore, modifications are needed to overcome the deficiencies associated with natural fibers.

**Figure 4:**
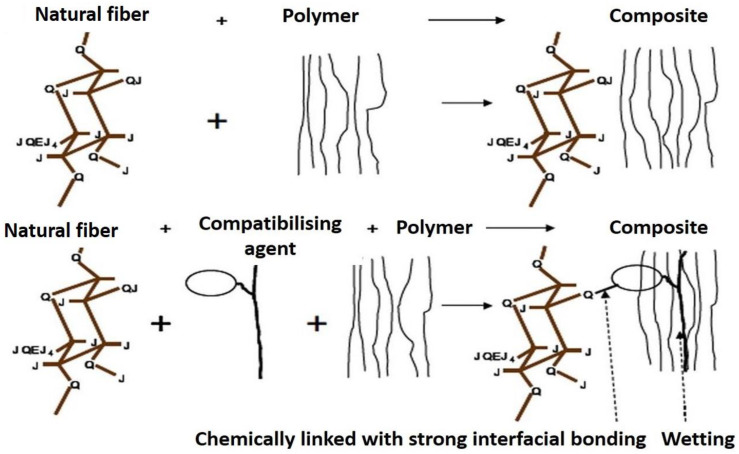
The coupling agent mechanism between the hydrophilic fiber and hydrophobic matrix polymer. Reproduced from ref. [43].

The modification toward natural fibers was used to alter the fibers’ physical, chemical, or morphological properties that prevent the natural hydrophilic fibers from bonding well with the hydrophobic polymer matrix. Several methods of modifications have been developed, including physical, chemical, and biological treatments. Chemical modifications are the most widely developed and effective in changing the structure of the fibers and improving the fiber–matrix interfacial bonding. Natural fibers can be treated via various types of chemicals such as alkali, silane, acetic acid, benzoyl chloride, potassium permanganate, acetic anhydride, and peroxides through this technique. These treatments were reported to enhance the mechanical properties of natural fibers by modifying the crystallinity structures and removing the weak components from the fiber surfaces [42]. The types of chemical modifications and their effect on the fiber composition and properties are discussed further. Upon chemical modification, the partial cementing structure components are broken and washed away, producing a clean and rough fiber structure. Figure 4 shows the coupling agent (i.e., silane) mechanism between the hydrophilic fiber and hydrophobic polymer matrix. In this case, the compatibilizing agent is chemically bonded to the fiber and blended by wetting in the polypropylene (PP) polymer chain [43].

### Effect on the Fiber Composition and Bonding Properties

The effects of chemical treatments on different types of natural fibers are listed in Table 1. In general, the function of chemical treatments is mainly to enhance the properties of the fibers by modifying their microstructure along with improvement in the wettability, surface morphology, chemical groups, and tensile strength of the fibers [44]. During the treatments, natural fiber impurities such as fats, lignin, and pectin were removed. The smooth surface and water absorption properties of the natural fibers are decreased, thus promoting better fiber–matrix interlocking, which leads to enhancement of the mechanical performance of the composites [42].

Many researchers have observed the effect of chemical treatments on the properties of natural fibers. Zafeiropoulos et al. [45] studied the effect of acetylation on different types of natural fibers, including flax, hemp, and wood fiber and reported removing non-crystalline fractions in fiber after the treatments. This led to an alteration of surface properties and changing the fiber surface energy, which improves the stress transfer properties at the interface. Hossain et al. [46] also reported a similar finding on alkaline-treated ladyfinger fiber. After the alkaline treatment, the interfibrillar region of the fiber is less dense and less rigid, allowing fibrils to re-arrange themselves along the direction of tensile loading. As the fibers stretched, the re-arranged fibrils resulted in better load sharing and increased stress development in the fiber. The study done by Latiff et al. [47] also proved the enhancement of fiber tensile strength by 47.5% after benzoylation treatment with 30 min of soaking time. This enhancement is attributed to removing lignin and other amorphous parts of the fiber, thus easing the fibrils to rearrange along the tensile direction and impart higher tensile strength. According to this, it is best to conclude that a significant improvement in the mechanical properties of the fiber and fiber composites can be achieved by using different chemical treatment processes. Table 1 shows the effect of chemical treatment of natural fiber-reinforced composites on the structural, mechanical, and thermal properties. 

## 3. Thermogravimetric Analysis (TGA)

Thermal analysis can be defined as a test used to evaluate chemical, physical, and structural changes in a material due to a temperature change. In principle, temperature is a fundamental state variable that affects most chemical reactions, physical properties, and structural transformations. As a general concept, thermal analysis can be defined as any scientific or technological characterization of a material in which temperature is varied as an experimental parameter. However, this term has long been limited to specific techniques related to thermogravimetric and calorimetric effects [64]. It is now widely accepted that the main techniques associated with a thermal analysis are the difference in temperature between a sample and a reference (DTA), the loss of weight measured by thermogravimetry (TG), its derivative (DTG), and the determination of heat flow by differential scanning calorimetry (DSC). Other techniques used to calculate the thermal conductivity, specific heat, and thermal diffusivity are, in many instances, embodied as thermal analyses. Different techniques or a combination of multiple techniques can be used when evaluating material characteristics depending on the purpose. Among these techniques, TG/DTG is one of the most sensitive thermal analyses extensively used to characterize the thermal stability of natural fiber polymer composites by analyzing their composition and structural dependency on thermal degradation behavior.

TG/DTG is an analytical technique used to determine the thermal stability and its fraction of volatile components by monitoring the weight change that occurs as a specimen is heated. On the other hand, TG/DTG is an experimental technique whose sample weight is measured as a function of sample temperature or time. Typically, the sample is heated at a constant rate. The weight is recorded as a function of increasing temperature and is normally performed in air or an inert atmosphere (such as helium or argon). The thermobalance on the TG/DTG instrument is used to measure weight change. To make the stable media and reduce the moisture, gas should flow during the experiment. Furthermore, buoyancy corrections in TGA measurements is required due to gas density changes. The sample will appear to show a mass increase during a heating experiment if buoyancy corrections are not applied. TG/DTG measurements are usually corrected for the effect of buoyancy by taking it as a control sample [65].

For sample preparation, the sample should be placed in a crucible. Thermograms are typically used to display TG/DTG results as curves of weight loss variation with temperature. As previously stated, lignocellulosic fibers are sensitive to temperature, and complete thermal degradation is expected above 400 °C [64,66]. Plants are mainly composed of cellulose, hemicellulose, and lignin, which is responsible for their fibers’ physical properties [67]. Other volatile or partially stable constituents, such as pectin, waxes, and water-soluble substances, may also exist in lignocellulosic fibers. As the major constituent, cellulose conditions the physical properties of natural fibers and has a significant contribution to their thermal degradation. The linear polymeric chain of the cellulose begins to decompose at relatively lower temperatures and, owing to a catalytic effect of naturally existing inorganic ions, may form a higher amount of char [68]. The hemicellulose found in plant cell walls comprises a diverse collection of polysaccharides that differs from plant to plant based on location and sources. It has a higher degree of chain branching than cellulose but a much lower degree of polymerization. Hemicellulose thermal degradation occurs before cellulose, but its content in the fiber proportionally limits its effect. Lignin is a complex hydrocarbon polymer with both aliphatic and aromatic constituents [67,69,70]. The thermal decomposition of lignin occurs in a broader range that initiates earlier but extends to higher temperatures than those of hemicellulose and cellulose degradation [71]. However, its effect is also limited by the smaller content in the fiber.

A natural fiber polymer composite is composed of two main parts, the natural fiber, as reinforcing phase, and the polymer matrix, which affects the thermal stability of the composite. Nurazzi et al. [3] stated TG and DTG curves for oil palm shell powder in the range of 35 to 900 °C. It can be seen that natural fibers usually degrade in three main stages with most of the thermal decomposition occurring in the range of 215 to 470 °C. The weight loss and DTG peak in the first stage can be attributed to water loss [72]. The thermal degradation of the main lignocellulosic constituents of the fiber begin to occur at the onset of the second stage. The third stage is decomposition of cellulose; the shoulder peak to the hemicellulose and the tail peak to the end of lignin decomposition. The weight remaining in the third stage could be assigned to char or other products from decomposition reactions. Hemicellulose is found to decompose at a maximum of 290 °C and up to 150 kJ/mol for activation energy, while lignin would thermally decompose with peaks from 280 to 520 °C and up to 229 kJ/mol for activation energy [73].

Another point worth discussing is the influence of different TGA atmospheres on the thermogravimetric results of natural fibers. In principle, two distinct atmospheres, inert (helium and nitrogen) and oxidative (air and oxygen), may be used. Moreover, as gases conduct heat at different rates, thermograms obtained in nitrogen may be significantly different from those obtained in helium. Under an inert atmosphere, the thermal degradation of cellulose results in a main DTG peak associated with the formation of macromolecules containing rings bearing double bonds. In an oxidative atmosphere, partial overlapping of this peak with the exothermic peak corresponding to the oxygen reacts with the cellulose. Consequently, the main DTG peak is shifted to lower temperatures in the oxidative atmosphere than the inert one. For instance, the maximum rate of decomposition of wood occurs at 320 to 330 °C in air and 350 to 370 °C in nitrogen [64,74,75].

## 4. Thermal Degradation Stability Performance of Natural Fiber-Reinforced Thermoplastic Composites

Thermoplastic composites originated from structural polymer composites, and they are chemically stable, since this kind of composite uses a thermoplastic matrix. There are many types of thermoplastics such as PP, polyethylene (PE), nylon, polystyrene (PS), polyvinyl chloride (PVC), poly (methyl methacrylate) (PMMA), and polytetrafluoroethylene (PTFE), some of which are presented in this section. Interestingly, these polymers can be molded into a variety of products. Thermoplastic polymers soften and can be remolded without degrading when heated, and they solidify into the final form when cooled. This heating–cooling cycle can be performed many times, providing the product almost limitless shelf life. They are also known as inexpensive, lightweight, and durable materials [76] which are useful for various applications, including consumer goods, machine parts, medical equipment, and packaging and storage materials. Scientists are continuing to explore several potentials to increase the performance of these thermoplastics’ polymers. Thermoplastic composites have the potential for large-series production of lightweight structural parts, saving weight and energy in future applications [77]. However, natural fiber as a less thermally stable material leads to critical issues for manufacturing natural fiber-reinforced thermoplastics composite products. For example, the thermal deformation of bathroom interiors can occur by the hot vapor generated during a shower. In addition, the car dashboard can be affected by the high temperature inside the vehicle during the hot season. Therefore, the thermal stability of these composite systems is critical because these materials must bear up against heat during a fire.

Many studies have reported on the chemical modifications of different fibers to be used in several thermoplastics composites to extend their applicability in the industry. The chemical treatment’s aim for natural fiber is mainly to improve the dispersion and compatibility between the natural fibers and thermoplastic polymers. The natural fiber is hydrophilic and the thermoplastics are hydrophobic. Thus, chemical treatment is usually applied to improve the interfacial bonding between these two different phases. Surface treatment using alkaline and coupling agents is the most common approach used in natural fiber-reinforced thermoplastic composites [78]. Alkaline treatment involves immersing fibers in a concentrated solution of NaOH. The NaOH reacts with the hydroxyl group of the natural fibers, removing some of the thermally unstable fiber constituents such as hemicellulose and lignin. These will increase the surface roughness, which improves interfacial bonding with thermoplastic polymer. Referring to Prasad et al. [79], the micrograph of the untreated fiber composite, in which fibers seem to be detached from the LDPE matrix and show relatively large pull-out and voids, indicating weak interfacial bonding between the fiber and matrix. In comparison, alkali treatment to the banana fiber provided relatively better interlocking between the fiber and the polymer matrix. This treatment increases the surface area contact by removing impurities, wax, and a portion of hemicellulose and lignin, resulting in fiber fibrillation and a rough surface. Furthermore, since thermally unstable fiber constituents (hemicellulose and lignin) is removed during alkaline treatment, the thermal stability of LDPE-reinforced alkaline fiber composites is much higher than that of untreated fiber composites.

**Figure 5:**
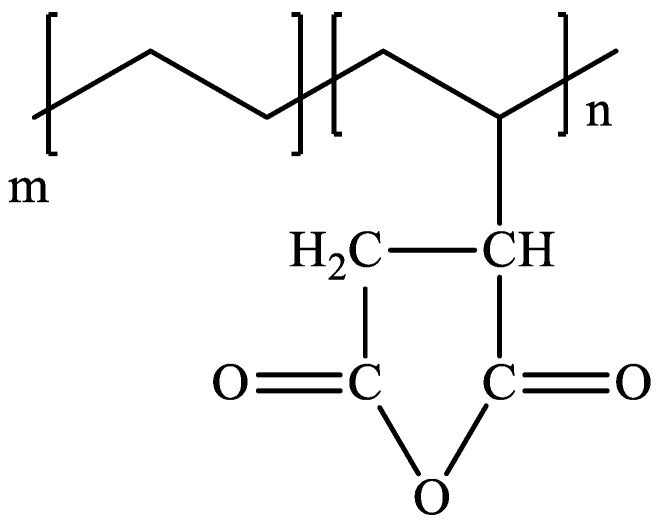
Chemical structure of MA-grafted PE.

Coupling treatment is another common chemical treatment applied on natural fiber-reinforced thermoplastics composites. This treatment aims to improve the weak interfacial compatibility between natural fibers and thermoplastic polymers. Several researchers concluded that grafted maleic anhydride (MA) had influenced the flexibility of the natural fiber-reinforced thermoplastics composite, thus proving that it functioned as a compatibilizer at the matrix interface [80,81,82,83]. MA functions at the interface to create a chemical bridge between reinforcement and the matrix by grafting onto polymer chains and forming bonds with natural fibers, giving a compatibilization effect. The result shows the dispersion of bagasse fiber in the thermoplastic polymer matrix was improved after the addition of 2 wt % MA-grafted PE. Figure 5 shows the chemical structure of MA-grafted PE. The absence of a coupling agent indicates a clear separation between HDPE and the fibers and a noticeable interface between HDPE with bagasse fiber [84]. Meanwhile, some fractured fibers emerged in the composite after the addition of 2 wt % MA-grafted PE, and the matrix was better bonded to the fibers, implying that the key energy dissipation mode in this scenario was fiber fractures rather than debonding. It can be inferred that MA-grafted PE substantially enhanced the interfacial adhesion between HDPE and fibers. In terms of thermal stability, the addition of MA-grafted PE had little influence on the degradation behavior of HDPE/bagasse fiber composites. Table 2 summarizes several findings on the effect of chemical or coupling agent treatments on the thermal degradation performance of natural fiber-reinforced thermoplastic composites. Several types of chemical or coupling agent treatments used were also highlighted in each composite.

## 5. Thermal Degradation Stability Performance of Natural Fiber-Reinforced Thermosetting Composites

Using natural fiber as a reinforced thermoset composite is very popular nowadays, as more parties look for an alternative sustainable composite material. However, the main disadvantage of natural fiber is its thermal degradation stability performance, combustible nature, and uncertain performance due to different parameters during cultivation. The importance of thermal degradation stability lies in the service condition, which will affect the performance of the composite material such as the glass transition temperature, char formation, and temperature of gasification of both matrix and reinforcement. Thermal degradation by thermogravimetric analysis normally occurs 3 times, which is the earliest at around 100 °C for water moisture evaporation, the middle at around 300 °C for the commencement of matrix decomposition, and the last, which is around 500 °C above for the total decomposition where char residue is fabricated.

Most researchers have suggested a step of pre-treatment on the kenaf fiber in order to improve several aspects of the fiber surface. Azwa and Yousif since 2013 had treated kenaf fiber with NaOH in order to increase the surface roughness. However, it appeared that by removing the lignin and hemicellulose on kenaf fiber, the thermal degradation became much faster, and a much lower moisture content was detected at 100 °C. On the other hand, char residue had increased by 14% on the treated kenaf fiber [91]. This is because alkalization removes lignin, which is responsible for charring; thus, untreated kenaf/epoxy composites contain more char than treated composites [92]. Asim et al. [93] had introduced a silane coating treatment on kenaf fiber/PALF with synthetic phenolic resin and found that a high temperature of thermal stability was successfully achieved and a high char residue of 20 to 35 wt % was left at 700 °C. There were two stages of thermal stability: the region of 250 to 350 °C, where kenaf fiber and PALF decomposed, and region 300 to 700 °C, where phenolic resin is degraded. PALF is another good natural fiber, as shown by researchers. In the last few years, several researchers fabricated PALF with phenolic resin in order to overcome the main weakness of natural fiber: its combustible nature. On top of that, they introduced silane treatment to the PALF with phenolic to improve the fiber surface roughness and discovered that its glass transition temperature increased [93]. This is supported by Eng et al. [94], who found that adding silane-treated fibers to composites leads to improvements in the fiber/matrix interaction. This is because strong bonding is required for the activation of the fiber constraint during the stress transfer mechanism at the interface; weak bonding leads to energy dissipation at the interface, and the impact of shifting is dependent on the fiber/matrix bond strength.

Bamboo fiber with phenolic foam resin cured at different temperature was tested in a research work carried out by Tang et al. It was made without fiber pre-treatment and found that an increase in fiber loading had increased the rate of thermal decomposition. Their thermal stability also improved due to phenolic foam undergoing a further curing process when the temperature increased during the analysis, thus augmenting its glass transition temperature as well. Phenolic foam only started to decompose by polymer chain scission at 450 °C and formed char residue of up to 67 wt % [95]. Another work by Chin et al. [96] about alkalization pre-treatment was done on bamboo fiber by varying the concentration of NaOH at different soaking times, which was topped with a physical milling process. The NaOH soaking had reduced the moisture content by reducing the hydrophilicity of the bamboo fiber. Increasing the soaking time led to a reduction of weight loss during the thermal decomposition phase, and the alkalization process had successfully increased the char residue by the formation of a lignin–cellulose complex [96].

Imoisili and Jen [55] had investigated the thermal behavior of KMnO_4_-treated plantain (*Musa paradisiacal*) fiber-reinforced epoxy composites. The concentration of KMnO_4_ used in their study is 0.025%, 0.05%, and 0.10%. The TGA result shows that the existence of a cellulose manganite complex was assumed to be accountable for the thermal stability of the KMnO_4_ treated with 0.025% and 0.05% fibers. This suggests that KMnO_4_ treatments have successfully oxidized the fiber and broken the hydrogen bond amid the -OH groups of cellulose and hemicelluloses. In contrast, at 0.10% of treated fibers, there was extreme oxidation resulting in cellulose degradation and a decrease in the thermal stability of the fiber [97]. This demonstrates that the KMnO4 treated samples with 0.025% and 0.05% have good thermal stability compared to those untreated and with 0.1% treated fiber.

Epoxy composites were prepared using acid-, base-, and silane-treated novel Caryota urens natural fibers, and their TGA was analyzed by Prakash et al. [98]. In this, 1 N concentration of sulfuric acid (H_2_SO_4_) and NaOH was prepared using the wet chemical method. The required quantity of Caryota urens fiber was immersed in the acid and base solution of 1 N for about 10 min and separated out. Similarly, the silane surface treatment was done using the aqueous solution method. The silane substance of 4 wt % was mixed with ethanol–water solution and mixed gently for 10 min of immersion. The TGA results show that the pure epoxy resin gives the initial, rapid, and final decomposition temperatures of 323 °C, 420 °C, and 492 °C. Further addition of untreated and surface-treated caryota urens in epoxy resin had reduced the thermal stability. The untreated fiber in epoxy resin offers a marginal decrement of 3.5% in the initial decomposition temperature. This reduction is the cause of the poor thermal stability of natural fiber, allowing evaporating the inbuilt moisture and the soft lignin. The addition of acid- and base-treated Caryota urens fiber in epoxy resin further reduces the thermal stability caused by the leaching phenomenon during the acid and base treatment process. The leaching could remove all thermal barriers in the outer shell of Caryota fiber. The thermal stability of untreated, base-, and acid-treated Caryota urens gives a larger mass loss than pure epoxy resin, whereas the composite reinforced with silane surface-treated Caryota urens fiber retains the thermal stability as equal to epoxy resin.

In conclusion, most of the fiber pre-treatments had successfully increased thermal stability with a more extended temperature range and higher temperature during degradation by coating the fiber to resist the thermal decomposition. This is a critical treatment process, as natural plant-based fiber is very susceptible to high temperatures. It is remarked that natural plant-based fiber will be decomposed first before the resin in the case of phenolic matrix, but for epoxy resin, if the natural fiber had high lignin content, this will affect their total weight loss at the end. As for the char residue quantity, if fiber pre-treatment involves removing lignin and hemicellulose from the fiber, this modification reduced char residue formation.

In conclusion, most of the fiber pre-treatment had successfully increased thermal stability with a longer temperature range and higher temperature during degradation by coating the fiber in order to resist the thermal decomposition more. This is an important treatment process, as natural plant-based fiber is very susceptible to high temperature. It is remarked that natural plant-based fiber will be totally decomposed first before the resin in the case of phenolic matrix but for epoxy resin, if the natural fiber had high lignin content, this will affect their total weight loss at the end. As for char residue quantity, if fiber pre-treatment involves removing the lignin and hemicellulose of the fiber, this modification reduced the formation of char residue.

## 6. Thermal Degradation Stability Performance of Natural Fiber-Reinforced Biopolymer Composites

Bio-based polymers are materials derived from renewable sources [99]. Bio-based polymers may be divided into three main categories based on their origin and production: Category 1: polymers directly extracted or removed from biomass. Examples are polysaccharides such as starch and cellulose, chitosan/chitin, and proteins such as casein and gluten [100]. Category 2: polymers produced by classical chemical synthesis using renewable bio-based monomers. A good example is PLA, bio-polyester polymerized from lactic acid monomers [101]. Category 3: polymers produced by microorganisms or genetically modified bacteria. To date, this group of bio-based polymers consists mainly of the polyhydroxyalkonoates (PHA), but developments with bacterial cellulose are in progress [102]. Biodegradable-based polymer has slightly polar oxygen atoms which could form hydrogen bonds to the hydroxyl groups of the natural fibers. However, from a literature review, it can be assumed that these hydrogen bonds only have a small influence on the fiber/matrix adhesion. Approximation between the polar groups cannot occur during processing because of the macromolecular structure of cellulose, hemicellulose, and lignin, which are the main components of natural fiber. Therefore, blending matrix and fiber caused fiber pull-out, problems in fiber dispersion, and poor adhesion properties of the composite [103,104].

However, some chemical treatment was carried out to overcome these drawbacks. The surface treatment of fiber is designed to facilitate a better adhesion between the fiber and the matrix. Surface treatment can be accompanied by either adding compatibilizers or by chemically treating the fiber to alter the polymer architecture [105]. Another challenge using natural fibers for composites is limited thermal stability. The processing temperature for natural fiber is limited to 200 °C [106]. Chemical treatment on fiber blending with bio-based polymer has a greater effect on the thermal properties of the composite. Table 3 showed the effect of fiber treatment on the thermal behavior of the composite.

The research on microfiber reinforced with PLA was carried out. The fiber was treated using enzyme modification by using NaOH in combination with a phosphate buffer and chelating agent. Enzymatic treatments have also been used to modify the physical network of natural fibers. The thermal properties of this composite were investigated by using heat deflection temperature. The deflection temperature results are useful to measure the relative service temperature for a polymer when used in load-bearing parts. At 348 °C and 386 °C, there was an increase in the thermal stability of the composite. However, the composite materials with 2 wt % of fibers exhibited an onset thermal degradation temperature (T_ons_) and degradation temperature (T_D_) practically identical to those of neat PLA. Only in PLA/SF-20, Tons, and TD were by 10 °C lower than for neat PLA. The analysis showed that fiber modification was improved minimally (2 to 4 °C). This could be due to a better interface between the fiber and the matrix. Therefore, the removal of unwanted materials on the fiber surface and some of the amorphous portions from the fiber surface were achieved without damaging the fiber surface when using the enzyme modification [109,110]. Since these enzymes eliminated the hemicellulosic portion of the fiber, George et al. [108] discovered that hemp treated with pectinmethylesterase (PME), xylanase, and xylanase + cellulase improved thermal breakdown. PME destroyed the pectic content of the hemicellulose structure, whereas xylanase degraded the xylan backbone. Furthermore, hemp fibers treated with polygalacturonase had poorer thermal stability, as shown by an increase in percentage degradation. This is due to the loss of polygalacturonan, which weakens the main cell wall, resulting in a weaker macrostructure.

Rosa et al. [107] studied the treatment of coir fiber incorporated with thermoplastic wheat starch. The coir fiber undergoes three types of different treatments: washing with water, alkali treatment, and bleaching. The thermal behavior of composites reinforced with treated fibers by water, alkali, and bleaching led to a positive effect on the thermal degradation behavior of the composites, as indicated by the higher thermal stability of the treated composites. For the treated-coir composites, the moisture loss peak shifted toward higher temperatures, from 134 °C for the untreated fiber composites to 142 °C, 143 °C, 145 °C, and 144 °C for washed, NaOH, and H_2_O_2_ fiber-reinforced composites, respectively. As reported in the literature, surface treatments also partially dissolved lignin and hemicellulose present in the fiber, increasing the amount of exposed cellulose; thus, coir fibers changed their crystallinity through alkali treatment [111]. The gradual elimination of amorphous hemicelluloses and lignin is associated with increasing crystallinity. NaOH solution may penetrate not only between crystallites but also inside crystallites, breaking inter- and intra-hydrogen connections between cellulose molecules and adjacent crystalline areas. As a result, the amorphous fraction is simpler to hydrolyze, reducing the overall quantity of amorphous cellulose while increasing the relative degree of crystallinity and crystalline index [112]. Many studies indicated that the thermal stability of composites is directly related to the crystallinity of the matrix and reinforcements, because high crystallinity can improve the heat resistance. It is noted that the introduction of NaOH treatment seems to improve the thermal resistance of treated fibers due the removal of the waxy layers and other impurities from the surface, as well as increased the crystallinity [113,114].

## 7. Thermal Degradation Stability Performance of Hybrid Natural Fiber-Reinforced Thermoplastic Composites

Modification of the fiber properties is expected to reduce the hydrophilicity and minimize the interfacial energy with hydrophobic polymer matrices, consequently improving the composites’ fiber–matrix interaction and appearance. Fiber hybridization is another approach that can be used to enhance the properties of the composites [115]. The hybrid fillers can be either natural/natural fibers or natural/synthetic fibers combination. Some important synthetic fibers that have been widely used include glass fiber, carbon fiber, aramid, and others. Hybrid fiber composites development aims to produce a product with performance characteristics that combine each component’s positive attributes. By mixing two types of fiber, one fiber could complement what is lacking in the other fiber [116,117]. Several works have been reported on treated and hybrid fiber composites by using natural fiber and synthetic fiber. A limited number of studies have been reported on natural fiber hybrid composites on thermoplastic polymers. By far, the most often studied thermoplastic polymers are PE and PP. Both of these polymers melt below 200 °C, making them suitable for the production of hybrid natural fiber composites.

A study was conducted on hybrid natural fiber using banana stem fiber (BSF) and coir fiber reinforced in the blend of maleic anhydride grafted polypropylene (MAPP) and LDPE. In this study, the BSF fibers undergo chemical modification involving bleaching, alkaline, and acetylation treatment. This step aims to reduce the hydrophilic component in the fiber, thus improving the fiber/matrix interaction. Hybrid composites were prepared with untreated, bleached, alkaline-treated, and acetylated BSF (0 and 10 wt %) combined with untreated coir fiber (5 wt %, 10 wt %, and 15 wt %) in the MAPP/LDPE matrix. The acetylated BSF/coir fiber hybrid LDPE composite demonstrates higher thermal stability for chemically treated fiber compared to the untreated fiber. They found out that the main degradation peak temperature shifted to a higher temperature (15 to 30 K) for treated fiber with a higher percentage of residues. This finding can be explained by the fact that the treatment reduced hemicellulose content in the fiber, thereby improving thermal stability [118]. This was also due to the acetylated fiber’s better compatibility with the MAPP/LDPE by the fiber/matrix intermolecular bonding.

Effects of surface modification of coir fiber and yam peel particulate by alkaline treatment on the properties of PP hybrid composite were conducted by Adediran et al. [119]. Both fibers can be categorized as untreated and treated fiber. The fiber was soaked in 1.5 M NaOH solution for 12 h at room temperature for coir fiber treatment, which was followed by washing with distilled water and sun drying. The fiber was prepared in a 30 mm length. Meanwhile, the YPP was treated in a 1 M solution containing a mixture of NaOH and HCl (70:30), washed with distilled water, and sun-dried. The dried peel was pulverized and sieved to about 45 μm particle size. The coir fiber (15 wt %) and YPP (2 wt %, 4 wt %, 6 wt %, and 8 wt %) were mixed with PP matrix by using a compression molding machine. Based on their finding, the incorporation of hybrid fibers enhanced the thermal insulation of the composites. They suggest that chemical treatment improved the properties of the composite by reducing the lignin and hemicellulose content in the fiber.

Sutradhar et al. (2018) studied the effect of fiber ratio and chemical treatment on the properties of banana and betel nut fiber-reinforced polypropylene hybrid composites. Both fibers were treated with 5% NaOH to remove impurities and other greasy content. Then, hybrid composites were prepared by varying the ratio of fiber added. The content of fibers was fixed at 15% with the banana and betel nut fiber composition added at 1:1, 3:1, and 1:3 ratios. This study shows that untreated fiber-reinforced PP shows better thermal stability than the NaOH-treated fiber composites. Based on the findings, an equal amount of fibers had the highest thermal stability than other compositions [120]. A study on banana/coir fiber-reinforced polypropylene hybrid composites was investigated by Gunturu et al. In their study, the banana fiber was extracted from banana stems and the coir fiber was obtained from coconut bunches. Both fibers were cleaned with water to remove waste residue and sun dried afterwards. The hybrid composites were prepared by the addition of different ratios of banana and coir fibers at 5/15 wt %, 10/10 wt %, and 15/5 wt %. A TGA thermogram shows a two-step decomposition stage for all ratios, which refers to the loss of moisture content and decomposition of the composites. The highest thermal stability was obtained at 5/15 (wt %) of banana to coir fiber content. The highest weight loss occurred at the 350 to 500 °C temperature range [121].

Further, another study was conducted by Jahan et al. on the effect of fiber ratio and chemical treatment of PALF and betel nut husk fiber on PP composites. Both fibers were chemically treated with 5% NaOH. For the untreated fiber, 10% fiber composite was prepared with different ratios of PALF and betel nut fiber (1:1, 1:3, and 3:1). Meanwhile, for treated fiber, the same composition was used with a 3:1 ratio of betel nut to PALF. Based on the thermal analysis study, it was evident that moisture loss occurred at below 100 °C for all samples. For the composite with a ratio of 1:1, the main thermal degradation stage occurred at around 250 to 410 °C, whereas for the ratio of 3:1, it happened at 290 to 450 °C. For the ratio of 1:3, the degradation occurred at 250 to 410 °C. On the other hand, for the composite containing alkali-treated fibers at a ratio of 3:1, thermal degradation occurred at a lower temperature at around 245 to 380 °C. From the outcomes, it was evident that alkaline treatment on fiber decreases the thermal degradation of the composites compared to the untreated fiber [122]. According to Yew et al. [48], untreated fiber has a larger moisture content than treated fiber, owing to the increased amount of hemicelluloses and lignin, resulting in more moisture absorption in the fibers. When untreated fiber is heated, there is greater weight loss due to the evaporation of moisture. Furthermore, the evaporation of moisture trapped in voids produced by the weak interface between the untreated fiber and the polymeric resin contributed to the weight loss of the untreated fiber composite at lower temperatures.

Hybrid fiber composites research on jute cellulose and bamboo cellulose with LDPE has been investigated [123]. The aim is to study the fiber treatment and fiber loading on the thermomechanical properties of the hybrid composites. Both fibers undergo chemical treatment of dewaxing, delignified, mercerization of fiber, and mercerization of cellulose—the composites prepared by using both untreated and treated fibers. The composite was prepared using a 1:1 ratio of jute to bamboo on untreated jute-bamboo/LDPE, jute cellulose/LDPE, bamboo cellulose/LDPE, and jute–bamboo cellulose/LDPE. Thermal analysis showed that the untreated fiber composites have an onset degradation temperature at 304 °C, whereas all of the treated fiber hybrid composites show an onset degradation temperature ranging from 346 to 415 °C. The high onset was due to the high thermal resistance of the treated fiber. From this, it can be confirmed that hemicellulose was removed in the treated fiber composites.

Moreover, based on the analysis, there is no significant difference in decomposition temperature for both untreated and treated fiber related to the major decomposition stage. The same group performs a comprehensive study on jute/bamboo fiber modified with a silane coupling agent and with the addition of a copolymer. The fiber was treated with 1% 3-aminopropyl triethoxysilane (APTS) to promote fiber/matrix interfacial adhesion. Both fibers were mixed at a weight ratio of 1:1. Then, 15 wt % of hybrid fibers with or without poly(ethylene-co-glycidyl methacrylate) (EGMA) (5 wt %) co-polymer were equally distributed on the LDPE laminate sheet to produce hybrid composites. Based on the thermal analysis study, degradation of the composites took place after 310 °C. The decomposition of untreated fiber composite was observed between 376 and 690 °C. The treated fiber displays the initial decomposition starting at 378 °C. The major decomposition was due to the degradation of hemicellulose, ɑ-cellulose, and LDPE resin. The addition of EGMA lowers the stability of the hybrid composites due to the low thermal resistance of EGMA-modified LDPE. The finding indicates that treated fiber composites possess better thermal stability. In the silane-treated fibers composites, the treatment provides better interlocking of the fiber with the matrix and, hence, higher thermal stability was achieved [124].

Another study on the effect of chemical treatment of jute fiber and sheep wool fiber has been reported. Jute fiber was chemically treated to increase its compatibility with sheep wool fiber and a polypropylene matrix. The alkaline treatment with 5% NaOH reduced the hydroxyl group, whereas diazonium salt treatment at acidic, neutral, and alkali media converted the hydroxyl group into the diazo group. Both treated and untreated fibers were used to prepare the hybrid composites. The total content of treated and untreated jute and sheep wool fiber was fixed at 15 wt % with a ratio of 3:1. The initial decomposition temperature of untreated and NaOH treated jute fiber is recorded at 218.9 °C and 215.5 °C. Meanwhile, the treated fiber at pH 3, pH 7, and pH 10.5 have initial decomposition temperatures of 215.3 °C, 223.0 °C, and 214.9 °C, respectively. The study found out that the neutral media of diazonium salt-treated jute fiber hybrid with sheep wool fiber composite has better thermal stability compared to the untreated fiber and other treatment [125]. According to the FTIR analysis, the diazonium salt decreased the hydroxyl group of cellulose anhydroglucose by breaking the OH group of carbon 6 and carbon 2 during the reaction and converting the two hydroxyl groups into diazo groups, resulting in an azo product, 2, 6-diazo cellulose [126]. The jute fiber, sheep wool fiber, and PP matrix all adhered better as a result of this occurrence. As a result, the particular composite’s tensile, flexural, and thermal characteristics improved.

Moving further, a hybrid composite of linear low-density polyethylene (LLDPE)/sugarcane bagasse/eggshell has been prepared. In this work, sugarcane bagasse undergoes silane treatment using 3 wt % of 3-(trimethoxysilyl) propyl methacrylate, while the eggshell was treated with 1 wt % titanium (IV) isopropoxide. The different ratios of sugarcane bagasse and eggshell were used to prepare hybrid composites in the range of 6/4 wt %, 12/8 wt %, 17/13 wt %, 20/20 wt %, 13/17 wt %, 8/12 wt %, and 4/6 wt %. Thermal analysis reveals that the hybrid composites have lower thermal stability than the neat LLDPE. All of the samples showed a major weight loss in the temperature range of 450 to 750 °C. The results also reveal that the thermal stability for both treated and untreated fiber decreased with the increasing fiber content. Based on the analysis, neat LLDPE shows a one-step degradation stage, while the hybrid composites have three decomposition steps. The first and second steps can be assigned to the decomposition of hemicellulose and cellulose fiber, whereas the third step corresponds to the complete temperature decomposition. In this case, the thermal stability of treated fiber composites was not significantly different from the untreated fiber [127].

Akter et al. [128] have performed a study on PALF/betel nut husk/PP. In this study, the fibers were cleaned using water and dried before usage. The fiber loading was varied at 5%, 10%, and 15%, while the fiber ratio was fixed at 1:1. The size for both fibers was around 3 to 5 mm in length. Thermal degradation for 5 wt %, 10 wt %, and 15 wt % fiber loading occurred at 225 to 358 °C, 250 to 410 °C, and 223 to 374 °C, respectively. Therefore, 10% fiber loading gave the highest thermal stability among the other composite samples. This can be explained by improving the fiber/matrix adhesion, as proven by SEM analysis. Kabir et al. [129] have summarized the factors that influence the properties of the composites, and they identified that fiber loading is one of the main factors that plays a vital role in determining the interfacial adhesion in a bonded fiber–matrix and the final properties of the composites. The right amount of fiber loading created better longitudinally aligned fiber; thus, the composites tend to achieve better interfacial adhesion. Better adhesion then promoted higher thermal stability of the composite due to the liquid–gas interface by the fibers. Table 4 displays the overview of the chemical treatment and hybrid fiber effect on the properties of various composite systems.

## 8. Thermal Degradation Stability Performance of Hybrid Natural Fiber-Reinforced Thermosetting Composites

Many studies have been reported to evaluate the thermal degradation and stability performances of treated hybrid natural fiber-reinforced thermoset polymer composites. Several experimental data and results on modified hybrid natural fiber thermosetting composites are presented in Table 5.

**Figure 6:**
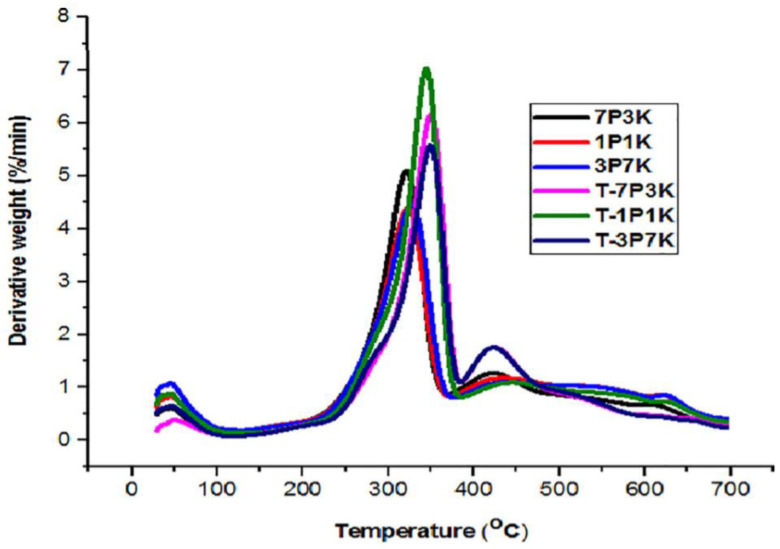
DTG curve of PALF/kenaf fiber hybrid biocomposites. Reproduced from ref. [93].

A study carried out by Asim et al. [93] discovered major weight loss of around 34 to 45% in the temperature range of 278 to 306 °C for untreated and silane-treated PALF/kenaf fiber hybrid composites. This observation occurred due to the breakdown of significant components of plant cells such as hemicellulose, lignin, and pectin, besides glycosidic linkages of cellulose [17,19]. As depicted in Figure 6, the DTG graph displays two distinct peaks, where the first peaks were below 100 °C due to hydroxyl groups in fibers. At the same time, the second peak was due to the manifestation of voids and loose fibers besides the degradation of hemicellulose and cellulose of the cellulosic fibers. Moreover, the results show that the weight losses of treated hybrid composites were elevated, which was possibly due to hemicellulose and lignin being partially removed during silane treatment. The silane coating also enhanced the overall thermal stability of the hybrid biocomposites [16]. Several studies have emphasized the benefits of utilizing silane as a natural fiber pre-treatment for composite manufacturing. In the presence of water, silane treatment produces a siloxane bridge that chemically connects the fiber surface and the matrix, and silanol is produced. Since both ends of the silanol react with respective cellulose hydroxyl groups and matrix functional groups, it offers molecular continuity across the composite interface [138]. Chad and Fahim [139] discovered that silane treatment enhances sisal fiber adhesion and moisture resistance by improving the wettability, mechanical characteristics, and water resistance of sisal–epoxy composites. In addition, silane treatment increases the interfacial interaction between bamboo fibers and rubber, influencing the mechanical characteristics of bamboo-reinforced polymer composites.

Apart from that, a comparison study between hybrid OPEFB fiber/jute-reinforced epoxy composites, OPEFB fiber/epoxy composites, and neat epoxy analyzes thermal stability and degradation properties [134]. Hybrid OPEFB fiber/jute fiber composites display enhancement in thermal stability after the addition of jute fibers. This is due to the breakdown of cellulose linkage; the initial thermal degradations of hybrid composites are located at a temperature range between 270 and 300 °C. Second thermal degradation can be found in thermograms at a temperature of 340 to 360 °C due to the breakdown of lignin [140]. The thermal degradation of hybrid composites was discovered at 250 to 400 °C due to the decomposition of the cellulosic and hemicellulose components. The finding also displays that hybrid composites elevate the temperature of thermal degradation due to jute fibers, which subsequently increase the final residue.

Another research work by Jawaid et al. [135] revealed that hybrid-treated OPEFB fiber/jute fiber/epoxy composites allow better thermal stability and degradation behavior. The thermogram result exposes that the initial weight loss happened at below 100 °C because of water in the fiber. At 250 to 450 °C, significant thermal degradation was found in this temperature range as the initial thermal degradation followed at 268 to 297 °C, which indicates breakage of α-cellulose and hemicellulose [141]. The temperature of final degradation was logged at 441 to 462 °C due to the complete breakdown of polymeric resin and lignocellulosic materials [142]. The mechanism of lignocellulosic thermolysis reactions was studied by Trindade et al. [143], and they concluded that the decomposition included the cleavage of glycoside bonds, CH, CO, and CC bonds, as well as dehydration. These processes formed carbon monoxide, carbon dioxide, and methane. Continuous heating leads to the saturation of the aromatic rings, the rupture of CC, the release of water, CO_2_, and CO, and structural rearrangements. This explains why hybrid composites exhibit thermal degradation within a larger temperature range. In addition, at higher temperature of more than 450 °C, the oxidation of the char was taken place, suggesting that the char has self-ignited, prolonging and stabilizing the degradation process [144].

A study conducted by Cavalcanti et al. [145] found out that there were two levels of thermal decomposition for jute fiber/sisal fiber-reinforced epoxy composites. A slight mass loss was initially observed at 110 to 150 °C due to water loss due to elevated temperature. Next, the second stage occurs during the pyrolysis process at 260 to 450 °C. The second stage of thermal decomposition also occurs because the main lignocellulose constituents were thermally degraded within this temperature range. Specifically, for the epoxy-based biocomposites, the degradation peak was higher i.e., 373 °C, which resulted from the chemical treatment of fibers. The treatment was found to aid in eliminated impurities and low-thermal-content components. As a consequence, the breakdown process occurs mostly in the cellulose part of the fibers, resulting in an increase in the alkalinized fiber-reinforced composites’ degradation temperature. Several studies discovered that chemical treatment may improve the material thermal stability by gradually removing non-cellulosic material such as hemicellulose and lignin [146,147]. Thus, alkaline-treated jute fiber/sisal fiber biocomposites would enhance degradation temperature.

The thermal degradation properties of hybrid jute fiber/glass fiber-reinforced epoxy biocomposites display 31% and 1.95% initial thermal degradation for jute and glass fibers, respectively [137]. At the end of the thermogram result, the weight loss of jute fibers in epoxy biocomposites was approximately 70.7%, while the residual weight percentage showed only 6.48%. The thermogram of 18% jute fiber and 19% glass fiber hybrid composites showed 1.27% weight loss. About 63.54% of final weight loss was discovered at 200 to 450 °C. Finally, the residual amount was only 24.19% of the original mass. From the research, as mentioned in earlier findings, the treated hybrid natural fiber composites enhanced the thermal stability and degradation. To be specific, the final thermal degradation temperature of treated hybrid natural fiber composites was elevated to a higher temperature. This happened due to a complex chemical reaction with treated compounds. This property of thermal degradation occurred due to the escalation of molecular weight caused by cross-linking between the polymer matrix and cellulosic material besides an extension of the molecular chain of the matrix itself [148,149].

## 9. Thermal Degradation Stability Performance of Hybrid Natural Fiber-Reinforced Biopolymer Composites

Biopolymers are polymers derived from natural origin and polymers produced by chemical synthesis that can be categorized into a few groups depending on the source of the biopolymers. Natural biopolymers are obtained in living organisms, mainly protein and polysaccharides such as collagen, silk, cellulose, and chitin. Meanwhile, synthetic biopolymers include biomass-based such as PLA and petroleum-based, such as polycaprolactone (PCL) and polyvinyl alcohol (PVA). In recent years, there has been a resurgence of interest in synthesizing composites from biopolymers sources. Numerous researchers have investigated the effects of the thermal behavior of natural fibers and biopolymers on the thermal degradation of composites. Starch, PLA, PVA, and PCL are the most popular biopolymers studied to manufacture panel products for composite applications [101,150,151,152]. However, a single natural fiber composite may not be the best option for achieving optimum properties to deal with synthetic fiber-reinforced composites. As a result, hybrid composites are created by combining two or more natural fibers or hybrids with synthetic fibers. Thus, a hybrid approach is advised to reduce moisture absorption, improving the mechanical properties while also improving the thermal properties of composites. In many reports, hybridization has been shown to increase the thermal properties of natural fiber-reinforced composites [153,154]. Table 6 lists some of the results on the properties of hybrid biocomposites from treated natural fiber reinforced with biopolymer.

A study on biodegradable starch-based hybrid composites comprising date palm and flax fibers was conducted by Ibrahim et al. [156]. Composites based on thermoplastic starch fiber-reinforced were fabricated using a compression molding and hot press process. In this study, date palm and flax fibers were treated using NaOH and corn starch as a matrix. The effects of fiber content (0, 20, 40, 50, 60, and 80 wt %) on their fiber properties, composite mechanical properties, and thermal properties were investigated. The results showed that the thermal stability increased as the fiber content increased, which was attributed to a rise in the volume of cellulosic content, which is more thermally stable than starch. This result seems to be in accordance with Averlous and Boquillon [164], who discovered that adding more cellulosic fiber to wheat starch-based biocomposites enhances their thermal resilience. Heat caused molecular chain ruptures, which started the degradation processes and the breakdown of the fibers and matrix structure. In general, increasing the degradation temperature onset and decomposition temperature of composites by adding high cellulose content material to the matrix enhanced the degradation temperature onset and decomposition temperature [165]. Improving the thermal performance of thermoplastic starch-based composites by increasing the date palm fibers means strong adhesion between the fibers and the TPS matrix. They also noticed that the temperature needed for 10% weight loss for date palm fibers composite increased from 192 °C for the starch-based matrix to 232 °C for the 50 wt % fiber composites.

Ramesh et al. [160] studied the hybridization of aloe vera fiber with filler, i.e., montmorillonite (MMT) clay, reinforced with PLA by using the compression molding method. In this study, aloe vera fiber was treated with 6% of NaOH solution for 3 h and used in the hybrid biocomposites fabrication with different ratios of MMT, namely 1, 2, and 3%. The outcome revealed that the hybridization improved the thermal stability of the composite, with a high proportion of the MMT clay in the hybrid PLA composite producing an optimum decomposition temperature, i.e., 350 °C. This was related to the presence of MMT clay that created a barrier effect by hindering the formation of small molecules, thus hindering chain mobility and delaying the decomposition phase. On the other hand, MMT clay acted as an insulator for mass transportation, increasing the thermal stability of volatile products produced in the decomposition [166].

In a different study, Ramesh et al. [160] researched the effect of MMT clay as a filler in the production of kenaf fiber-reinforced PLA composite. The kenaf fibers were treated with 6% of NaOH solution for 3 h, and the amount of MMT used was 1, 2, and 3%. The result shows that the trend in the thermal decomposition of the hybrid kenaf fiber composite was similar to the trend in hybrid aloe vera composite, where the presence of MMT clay improved the thermal stability. The MMT clay acts as a barrier, preventing the PLA polymer matrix from volatilizing. The segmental mobility of the polymer networks between the clay layers is limited, resulting in better thermal stability characteristics. Furthermore, the inclusion of high temperature-resistant moieties in the MMT clay, such as the triphenylphosphine unit, contributed to the composites’ improved thermal stability [167]. In addition, the heat resistance index of the hybrid composite containing 3% MMT was the highest, 153 °C, compared to those of 1 and 2% MMT, which were only 144 and 149 °C, respectively. The treated fiber also improved the thermal stability due to the better compatibility between PLA and MMT of PLA/kenaf fibers/MMT hybrid biocomposites.

**Figure 7:**
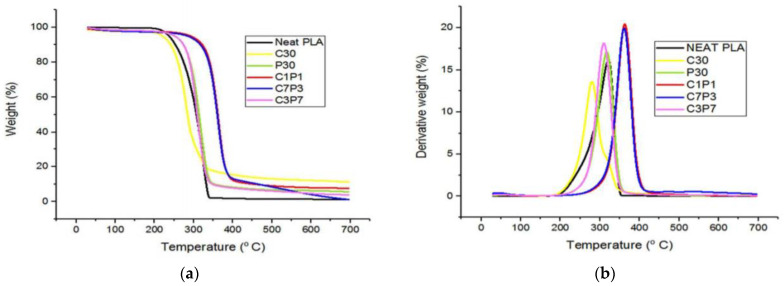
The (**a**) TGA curve and (**b**) DTG curve of coir and PLAF fiber hybrid PLA composite and neat PLA [168].

Several studies have been conducted on the thermal degradation properties of different hybrid natural fiber-reinforced biopolymer composites. Siakang et al. [168] investigated the thermal stability of PALF hybrid with coir fiber by using PLA as a matrix. The effect of the different amounts of coir and PALF ratios, namely 1:1, 3:7, and 7:3 reinforced hybrid PLA composites, on the resulting thermal behavior was studied. They found that the hybrid PLA composite with more PALF elevated the thermal properties, as indicated by the TGA and DTG curve in Figure 7. The composite’s degradation temperature contains PALF associated with the chemical component, where PALF has lower lignin content, and coir fiber has low cellulose content. Higher lignin content was found to improve the thermal performance due to the fact that lignin decomposition may occur at temperatures ranging from 200 to 700˚C [169]. They also concluded that hybridizing the same weight ratio of both fibers resulted in optimum thermal properties of PLA composites.

In another study, both fibers were treated with NaOH solution for 3 h and used in the hybrid composite production with the same fiber ratio [161]. It was observed from the TGA analysis that the alkaline treatment resulted in the increments of weight loss temperature and reduction in Tg temperature. Alkaline treatment is associated with modifying the fiber surface by removing a specific rate of lignin, hemicellulose, wax, and oils covering the fibers’ surface and partially dissolved non-cellulosic components fiber. The breakage of ester bonds between lignin and polyuronic caused by alkali treatment partly dissolved the binding material, thus increasing the crystallinity index in alkali-treated fibers. This led to an improvement in the amount of exposed cellulose, altering the crystallinity of the treated fibers [170,171]. Higher crystallinity was found to increase the tensile strength of the composite due to the higher compatibility between the fibers and matrix. However, from the DTG analysis, they found that the weight loss was minimal in the untreated hybrid composites, indicating that the composite showed good thermal stability due to fewer unreacted components. They concluded that the thermal stability of the composite is affected by treatment, but the difference is minimal, and the thermal expansion value of treated hybrid composites was lower than that of untreated hybrid composites.

Zhu et al. [172] produced hybrid composites from untreated and alkali-treated sisal fibers with a PLA matrix to produce hybrid PLA–sisal fiber composites. Zhu et al. observed that the enhanced impact of the hybrid composite was more significant than that of the treated composite. The impact strength and crystallinity of the hybrid composite had apparent changes. Compared with neat PLA composite, there was a slight reduction in PLA–sisal and hybrid composite thermal stability due to the fibers and PLA experiencing heat and friction during processing, resulting in thermal degradation. However, that PLA–hybrid sisal composite had a higher initial pyrolysis temperature, final pyrolysis temperature, and maximum pyrolysis rate temperature than PLA–sisal fiber, indicating the more excellent thermal stability of the hybrid composite because of a better chemical structure and higher crystallinity.

## 10. Conclusions

Natural fiber-reinforced polymer composites have gained increased attention among researchers due to their low density, specific strength, biodegradability, etc. Despite these advantages, the hydrophilic nature, i.e., low moisture resistance and poor fiber/matrix adhesion characteristics of natural fibers, has been a serious drawback to their application in various industries. Through chemical treatments to improve the limitations mentioned earlier, the reported literature has attempted fiber surface modification. The most commonly used chemical methods include alkalization, acetylation, benzoylation, and silane treatment. Among the various chemical treatments, alkali treatment seems to be the most economical and effective method for improving moisture resistance, wetting characteristics, and interactions with the hydrophilic nature of natural fiber with hydrophobic polymer matrix and synthetic fibers.

The alkalization of natural fibers leads to the fibrillation effect, splitting a single-fiber bundle into smaller ones, increasing the effective area for mechanical interlocking between fibers and matrix: thus, leading to improved interfacial bonding that finally improved the thermal stability and shelf life upon degradation of the composite structures. Improvement and deterioration of the alkaline-treated natural fibers’ thermal stability as well as that of the composite depend heavily on the concentration level of the chemical treatment, fiber loading, and immersion time. Thus, there exists an optimum percentage of concentration level, fiber loading, and immersion time for every natural fiber and its composite beyond which those properties face a detrimental effect, as reported by several researchers. In general, TGA results of weight loss percentage with increasing temperature showed two to three stages of characteristics depending on the natural fiber, chemical treatment conditions, and immersion time. The first phase involves the evaporation of moisture, and the second phase consists of the removal of cementing substances, which corresponds to the degradation of amorphous structures of hemicellulose, semi-crystalline of cellulose, and lastly, the degradation of lignin. The onset degradation between each natural fiber may be different even after the chemical treatment. This is due to the chemical composition of the natural fiber itself to prolong and maintain the thermal stability upon the degradation rise up.

The polymer matrix decomposes in between the range of 300 and 500 °C. Either for structural or non-structural applications, other potential strategies for the thermal stability improvement for composite structures with chemically treated natural fiber-reinforced polymer composites are hybridizing with synthetic fibers and carbonaceous materials. Thermal stabilizers from inorganic compounds such as metallic hydroxide additives are preferred due to environmental and health safety reasons, with MMT, CNTs, and graphene showing outstanding performance in term of thermal stability.

## Figures and Tables

**Table 1:** Chemical treatment of natural fibers and effect on structural, mechanical, and thermal properties.

Natural Fiber	Polymer	Chemical Treatment	Effect on Properties	Ref.
Coir fiber	Epoxy	Alkaline	The fiber surface roughness becomes significant as the duration of NaOH treatment increased The removal of hemicellulose and lignin was proved by FTIR analysis Better thermal stability at a lower temperature Lower water absorption for the treated sample	[48]
Kenaf fiber	Epoxy	Alkaline	Treated fiber is shown to have an increment in tensile strength and surface roughness but decreases in diameter IFSS properties of composites increased by 10.34% after being treated with 2% alkali	[49]
Kenaf fiber	Unsaturated polyester	Alkaline	Alkaline treatment using 5% NaOH solution Increment of 15% tensile strength, 20.7% flexural strength, and 17.89% impact strength	[50]
Cotton	-	Silane	Five different silane coupling agents were used to treat cotton fiber APTES showed the lowest moisture absorption, highest tensile strength, and better thermal stability among all silane-treated cotton	[51]
Sugar palm fiber	TPU	Alkaline + Silane	Treatment of 2% silane roughens the surface of the fibers and promotes mechanical interlocking with the TPU matrix The combined alkaline and silane treatment improved properties less in terms of physical, tensile, and IFSS properties	[52]
Flax fiber (woven)	Bio-epoxy	Silane	ILSS test showed 7% improvement in the interaction between the fiber and matrix of silane-treated composites assisted with the oxidation process. Water absorption was significantly reduced by 20% for oxidized silanized fiber-reinforced composites	[53]
Flax fiber	PLA	Silane	Decrease in damping properties due to the formation of the immobilized macromolecular chain due to strong interaction at the interface Morphology study depicted the crack propagation and shows a cohesive interfacial failure at higher load, indicating enhanced load transfer from PLA to the flax fiber	[54]
Plantain fiber (*Musa Paradisiacal*)	Epoxy	Permanganate	Enhanced thermal stability, crystallite size, degree of crystallinity, and inherent reduction hydrophobicity of the fiber Enhanced bonding between the fiber/matrix thus imparts enhanced mechanical and water resistance	[55]
Coconut fiber	Unsaturated polyester	Alkaline + Permanganate	Tensile strength decreases after fiber is treated with combined alkali and permanganate treatment Alkaline treated alone showed better fiber ILSS	[56]
Sugar palm fiber	TPU	Permanganate	Highest tensile strength with a concentration of KMnO4 of 0.125% with 6% alkali pre-treatment Fracture surface showed fibers stay intact within the matrix, indicating superior bonding properties	[57]
Bamboo fiber	-	Acetylation	Prediction via Taguchi and genetic algorithm showed acetylation able to reduce waster absorption up to 151%	[58]
Bagasse fiber	PE	Acetylation/Alkali/Stearic acid	Acetylated treatment upon bagasse fiber showed better compatibility toward PE by showing the highest mechanical and better water absorption resistance than that of alkali and stearic acid-treated samples	[59]
Raffia fiber	Unsaturated Polyester	Alkali/Acetylation	Acetylated fiber improves the tensile strength whilst alkali-treated give better modulus of elasticity and extension at break Acetylated fiber composites at 5% loading showed better fiber–matrix interfacial bonding compared to alkali-treated fiber composites	[60]
Jute fiber	PLA	Alkali/Benzoylation/Sodium bicarbonate	Benzoyl chloride-treated PLA coated jute composite with improvement in tensile and flexural strength, storage and loss modulus, and glass transition temperature Alkali-treated sample showed the highest impact strength	[61]
Teak sawdust	HDPE	Benzoyl peroxide	Improve in mechanical and water absorption test for all treated peroxide sawdust–HDPE composites SEM images proved the improvement in interfacial adhesion for treated sawdust–HDPE composites	[62]
Cereal straw	NR	Benzoyl peroxide	Better thermal resistance up to 220˚C. Reduction of tangent delta value indicates improvement in fiber–matrix interfacial adhesion	[63]

IFSS: Interfacial shear strength; ILSS: Interlaminar shear strength; TPU: Thermoplastic polyurethane, PE: Polyethylene; PLA: Poly (lactic acid); HDPE: High-density polyethylene; NR: Natural rubber; KMnO_4_: Potassium permanganate.

**Table 2:** Thermal degradation performance of chemically and coupling agent treated natural fiber-reinforced thermoplastics composites.

Polymer Composite	Chemical/Coupling Agent Treatment	Findings	Ref.
PP/Coir fiber	Chemical treatment: Cr_2_(SO_4_)_3_.12(H_2_O), NaHCO_3_ and HCl	− The chemically treated coir fiber composites is more thermally stable than the untreated fiber composite.	[84]
HDPE/Bamboo fiber	Coupling treatment: MA	− The decomposition temperature ranges of untreated bamboo fiber reinforced HDPE composite at 20 wt % fiber loading was lower as compared to neat HDPE. This was due to dehydration from cellulose unit and thermal cleavage of glycosidic linkage by transglycosylation and scission of C-O and C-C bonds. − The MAPE-treated bamboo composite at 2 wt % MAPE concentration displayed higher decomposition temperature than that of the untreated bamboo composites. This ensures a higher thermal stability in the composites treated with MAPE.	[85]
HDPE/Natural fibers (pine, bagasse, rice straw, and rice husk)	Coupling treatment: MAPE and MASEBS	− All natural fiber reinforced HDPE composites have lower thermal stability as compared to neat HDPE. − The addition of 2 wt % MAPE and 5 wt % MASEBS had only little influence on the decomposition behaviour of HDPE/bagasse fiber composites.	[86]
LDPE/Banana fiber	a. Chemical treatment: Alkali (1% NaOH) and acrylic acid (1%) b. Coupling treatment: LDPE functionalized with MA-g-LDPE	− The onset and final degradation temperature values of the LDPE matrix decreased with the addition of the fibers. This is owing to the thermal stability of the banana fiber which is much lower than that of the pure LDPE matrix. − Chemically treated fiber composites are more thermally stable than the untreated fiber due to the removal of thermally unstable fiber constituents (hemicellulose and pectin). − The compatibilizer (3 wt % MA-g-LDPE) seems to have little impact on the thermal degradation of composites. The addition of MA-g-LDPE to an untreated fiber composite increased both the onset and final degradation temperatures. Contrarily, MA-g-LDPE induced a decrease in the onset temperature value for the treated fiber composites. This is due to the addition of MA-g-LDPE improved the interfacial interaction between the fibers and the LDPE matrix by generating strong ester linkages between them.	[79]
PET/Flax fiber	Functionalization with nano (micro) particles of titanium or alumina	− The thermal degradation performance of layered composites based on PET/functionalized woven flax fiber show significantly improved values compared with the control sample (PET/not functionalized flax fiber). − Improved thermal degradation performance is due to links developed at the woven flax fiber/polymer phase interphase.	[87]
PVC/Natural fibers (bagasse, rice straw, rice husk, and pine fiber)	Impact modifier by using SEBS	− Incorporation of natural fibers lowered the thermal stability of PVC/natural fiber composites compared with neat PVC. − The addition of 2.5 wt % and 5 wt % SEBS had little effect on the thermal stability of the PVC/natural fiber composites.	[88]
PS/Agave fibers	Graft copolymerization of MMA	− PS composites reinforced with MMA grafted Agave fibers have higher decomposition temperatures as compared to untreated fiber reinforced composites. − The higher decomposition temperatures of composites reinforced with grafted fibers may be attributed to better fiber/matrix adhesion achieved as a result of surface modification which further increases the thermal stability.	[89]
Nylon/Natural fibers (coconut shell particles, empty fruit bunch fibers)	Chemical treatment: Alkali (5% NaOH)	− Thermal degradation of the composites was observed to start at lower temperatures than the neat nylon, particularly when EFB fibers were loaded at 15 wt %. − However, thermal degradation with treated fibers is higher as compared to the untreated fiber composite.	[90]
TPU/Kenaf fiber	Chemical treatment: Alkali (2, 4, and 6% NaOH)	− The TPU/kenaf fiber composites decompose at a temperature less than neat PU. − Thermal degradation performance showed that untreated TPU/Kenaf fiber composite was more thermally stable than treated composites, indicating that the interaction of fiber and matrix decreased after NaOH treatment. − The untreated composite has a less mass loss in the temperature range between 300 °C and 500 °C than all treated composites.	[78]

HCl: Hydrochloric acid; MAPE: Maleated polyethylene; MASEBS: Maleated triblock copolymer styrene–ethylene/butylene–styrene; MA-g-LDPE: Maleic anhydride-grafted LDPE; MMA: Methyl methacrylate; SEBS: Styrene–ethylene/butylene–styrene; Chromium sulfate (Cr_2_(SO_4_)_3_.12(H_2_O)), sodium bicarbonate (NaHCO_3_) and HCl.

**Table 3:** Effect of fiber treatment on the thermal properties of composite.

Natural Fiber	Polymer	Treatment	Thermal Stability	Ref.
Coir	Thermoplastic starch	NaOH: 10% *w/v*	Maximum weight loss at 317 °C	[107]
Coir	Thermoplastic starch	Bleaching treatment: H_2_O_2_, NaOH (30% *w/w*)	Maximum weight loss at 343 °C	[108]
Cellulose nanofiber	PLA	Aqueous solution of N-Methylmorpholine (20% *w/w*)	Decrease thermal desorption by 10 °C	[109]

**Table 4:** Overview of the chemical treatment and hybrid fiber effect on the properties of various hybrid composite systems.

Polymer	Natural Fiber	Treatment	Effect on Properties	Ref.
MAPP/LDPE	Banana stem fiber/Coir fiber	Bleaching, alkaline treatment, and acetylation	− Lignin was removed during the bleaching process. − Fibrillation took place in a part of the fiber because of the removal of the cementing material (lignin) and hemicellulose. − Alkali treatment increases the surface roughness and thus resulted in better mechanical interlocking. − Chemical treatment on fibers improves their thermal stability.	[118]
PP	Coir fiber/Yam peel	Alkaline treatment	− Chemical treatments on the fibers reduced the lignin and hemicellulose content, which resulted in the properties improvement of the composites. − Reinforcement of coir fiber and yam peel particulate into PP improved thermal insulation of the composites.	[119]
PP	Banana/Betel nut fiber	Alkaline treatment	− Alkaline treatment increases fiber surface roughness, resulting in better mechanical interlocking. − Higher content of banana fiber showed better mechanical properties compared to higher betel nut fiber composite. − An equal amount of fibers added gives the best thermal stability in a hybrid composite.	[120]
PP	Banana/Coir fiber	Washing with water	− The mechanical properties of hybrid composites were affected by fiber orientation, fiber length, and distribution of fiber. − Fiber ratio plays a significant effect on the thermal stability of the hybrid composites.	[121]
PP	PALF/Betel nut husk fiber	Alkaline treatment	− NaOH treatment of fibers decreased the tensile and flexural properties of the composites but increased their hardness. − NaOH treatment on fibers resulted in partial dissolution of amorphous phases waxes, hemicelluloses, pectines, and lignin). − NaOH treatment on fibers decreases the thermal stability of the composites compared to the untreated fiber.	[122]
LDPE	Jute cellulose/Bamboo cellulose	Dewaxing, Delignification, Mercerization	− FTIR study reveals the removal of hemicellulose and lignin after the fibers underwent chemical treatment. − All treated fiber composites have significant high mechanical properties compared to untreated fiber. − Hybrid composite with 10 wt % fiber loading shows the highest tensile strength and Young’s modulus. − No significant difference on decomposition temperature for both untreated and treated fiber hybrid composites related to major decomposition stage.	[123]
LDPE	Jute/Bamboo	Amino silane treatment and EGMA	− FTIR spectra of treated jute–bamboo hybrid composites revealed effective removal of -OH groups from cellulose using APTS. − The tensile strength and Young’s modulus of the treated hybrid composites 150% and 330%. − Treated fiber composites show better thermal stability due to better fiber/matrix adhesion. − Reduction in the crystallinity index is an indicator on the improvement of the fiber/matrix adhesion.	[124]
PP	Jute/Sheep wool fiber	Alkaline treatment and o-hydroxy diazonium chloride treatment	− Elimination of hemicellulose content after the alkaline treatment was proven by FTIR analysis. − Hybrid PP composites containing neutral media diazonium salt-treated jute fiber and untreated wool fiber has the best thermal stability among all prepared composites.	[125]
LLDPE	Sugarcane bagasse/Eggshell	Silane treatment; eggshell was treated using titanium (IV) isopropoxide	− Surface-treated fiber composites showed better dispersion and embedded within the LLDPE matrix compared to the untreated fibers. − Hybrid composites have lower thermal stability than the neat LLDPE.	[127]
PP	PALF/Betel nut husk	Washing with water	− Tensile strength decreased with an increase in fiber loading. − Flexural strength increased with an increase fiber loading. − 10% fiber loading gave the highest thermal stability among other composite samples.	[128]
PP	Sisal fiber/Glass fiber	Alkaline treatment	− Tensile, flexural, and impact properties of the composites increased by the addition of compatibilizer due to the improvement of fiber/matrix interfacial adhesion. − Addition of glass fiber improved thermal stability. − Thermal stability increased with increasing glass fiber content.	[130]
HDPE	Kenaf fiber/CaCO_3_ Rice husk/CaCO_3_	-	− Thermal stability of hybrid composite filled with kenaf fiber or rice husk is better than unfilled CaCO_3_/HDPE. − CaCO_3_/HDPE composite shows two decomposition peaks, while the hybrid composites show three decomposition peaks. − For the hybrid composites, the first peak around 300 to 400 °C is attributed to the decomposition of natural fiber, whereas the second and third resulted from the decomposition of HDPE and CaCO_3_.	[131]
TPU	Sugar palm fiber/Glass fiber	Alkaline treatment, silane treatment and combined alkaline–silane treatment	− Alkaline treatment removed impurities and hemicellulose. − Silane treatment reduced the hydroxyl group at the surface of sugar palm fiber by inducing a hydrophobic silane group. − The combine treatment showed a good result of lower density, thickness swelling, and low water uptake than the single treatment. − Improved thermal stability for the combined treated sugar palm composite compared to the untreated fiber.	[132]
PP	Sisal fiber/Halloysite nanotubes	Alkaline treatment and high intensity ultrasound	− Alkaline and HIU treatments of sisal fiber enhanced the interaction between sisal fiber and silane-grafted HNT, thus improving the interfacial adhesion between the filler and PP matrix. − The surface-treated sisal hybrid fibers composite showed an increased in thermal stability up to 40 °C increment with the addition of HNT. − This is attributed to the effective filler dispersion and compatibility between the polymer matrix and surface-treated hybrid fillers.	[133]

**Table 5:** TGA of natural fiber-reinforced hybrid thermoset polymer composites.

Polymer	Natural Fiber		Treatment	T_D_	Ref.
Phenolic	70% PALF	30% Kenaf fiber	2% silane for 2 h	402	[93]
Epoxy	33% OPEFB fiber	67% Jute fiber	10% benzoyl peroxide	441	[134,135]
Epoxy	50% Jute fiber	50% Sisal fiber	5% of NaOH solution for 30 min	517	[136]
Epoxy	25% Jute fiber	7% Glass fiber	Alkaline treatment	450	[137]

**Table 6:** Natural fiber-reinforced hybrid biopolymer composites.

Biopolymer	Natural Fiber	Ref.
Thermoplastic starch	Date palm fiber + flax fiber	[155]
Corn starch	Cornhusk fiber + sugar palm fiber	[156]
PLA	Sisal fiber + coir fiber	[157]
PLA	Kenaf fiber + nano clay	[158]
PLA	Aloevera fiber + nano clay	[159]
PLA	Kenaf fiber + nano clay	[160]
PLA	Coir fiber + PALF	[161]
Thermoplastic sugar palm starch agar	Sugar palm fiber + seaweed	[162]
Poly (butylene succinate) (PBS)	OPEFB fiber + tapioca starch	[163]

## Data Availability

Not applicable.
